# Complicated Neuraxial Anesthesia in a Patient With Tethered Spinal Cord

**DOI:** 10.7759/cureus.18705

**Published:** 2021-10-12

**Authors:** Eitan A Scher, Sabry Ayad

**Affiliations:** 1 Outcomes Research, Cleveland Clinic, Cleveland, USA; 2 Anesthesiology, Cleveland Clinic, Cleveland, USA; 3 Anesthesiology, Cleveland Clinic Fairview Hospital, Cleveland, USA

**Keywords:** epidural anesthesia, anesthesia and pain, procedure-specific anesthesia, obstetrics and gynecology anesthesia, combined spinal epidural, tethered spinal cord

## Abstract

Spinal anesthesia is a common anesthetic used in many surgical procedures. Anatomical variations and diseases make this procedure require extra considerations. Extra care should be taken into consideration in the pre-anesthesia evaluation when neuraxial anesthesia is planned.

We present the case of a patient undergoing cesarean section for the arrest of labor, who had a complicated labor course due to unrecognized history of a tethered spinal cord leading to inadequate analgesia, requiring replacement of epidural analgesia and spinal anesthesia. Ultimately, the decision was made to proceed with general anesthesia due to the patient’s discomfort. Exhaustive chart review after surgery showed a past MRI with evidence of a tethered spinal cord.

## Introduction

Neuraxial anesthesia requires precision to accurately block the spinal segmental level innervating the intended anatomical structures. Taking into consideration the fragility of the spinal cord, anticipating potential complications during the procedure, like anatomical variances or puncturing the incorrect structures, are vital to avoid permanent neurological deficits.

Tethered spinal cord syndrome is a disease in which the usually mobile and flexible spinal cord is anchored to the spinal canal, which is an immobile structure. Because of this, actions like bending over will ultimately stretch the spinal cord causing a stretch-induced functional disorder. This disease is part of a larger group called closed spinal dysraphism, which develops during gestation. Open or closed spinal dysraphism occurs at a frequency of 0.5 to 8 cases per 1000 live births [1]. Of those, tethered spinal cords are even rarer estimated at 0.25 per 1000 births [2]. Since this is a fairly rare disease, most patients are missed or undiagnosed. We present a case of a patient with tethered spinal cord and discuss the importance of accurate preoperative evaluation and documentation.

## Case presentation

This is a case of The American Society of Anesthesiologists (ASA) class 2, 24-year-old female G2P0010, with a height and weight of 170.2 cm (5’7”) 68.2 kg (190 lb), respectively, who presented to the obstetrics service at 38 weeks after having abdominal cramps overnight. Her pregnancy was uneventful. She was adherent with the prenatal appointments and investigations, which were unremarkable. According to her pre-anesthesia evaluation, the patient indicated that her past medical history consisted only of anxiety, depression, and tonsillectomy. Her obstetric history was remarkable for a spontaneous abortion before this current pregnancy. During the initial evaluation, the physical exam was reported within normal limits, with normal back observations without any evidence of skin dimple or unusual tuft of hair. Specifically, evaluation and questioning about back pain, bladder dysfunction, leg weakness, calf muscle atrophy, diminished or absent deep tendon reflexes, and dermatomal sensory loss were negative.

The patient was found to have positive anti-E antibodies. Thus, induction of labor was indicated and staff followed the standard of care protocol. She was offered labor analgesia which she accepted. An epidural block was placed at interspace L3-L4, the catheter was inserted while the patient was in a sitting position at a depth of 12 cm on the second attempt. There was no blood or cerebrospinal fluid (CSF) on aspiration and, test dose was negative and no paresthesia was noted. The epidural infusion consisted of bupivacaine 0.0625% fentanyl 2 mcg/mL-epinephrine 1.25 mcg/mL at a rate of 9 mL/h. The patient attained complete analgesia evidenced by a decrease of self-reported pain from 8/10 to 0/10 (by visual analog pain score where 10 is the worst pain and 0 is no pain) before and after catheter insertion, respectively, achieving a sensory block up to the level of T6. During the placement of the epidural catheter, fetal heart rate was 110 bpm, with moderate variability and present accelerations. The patient’s sensory perception was again assessed 10 hours after infusion started and revealed a decrease in the height of the block down to the level of T12; the epidural infusion rate was increased to 10 mL/h. Four hours after the infusion rate was increased, the patient complained of discomfort which progressed despite receiving several top-off bolus doses of epidural local anesthetic with 5 ml bupivacaine 0.25% or 5 ml lidocaine 2%. The epidural catheter was then replaced with a combined spinal-epidural block while the patient was in the sitting position. The replacement procedure was uneventful, 3.75 mg (=0.5 mL) of bupivacaine 0.75% was injected intrathecally. The new epidural catheter was placed on the first attempt at interspace L2-L3. Again, the test dose was negative, there was no blood nor CSF aspiration or complaints of paresthesia. Sensory evaluation after the procedure revealed a successful block up to the level of T6. Surprisingly, at this point, she still remained with high-intensity pain in her mid-lower abdomen despite multiple epidural local anesthetic boluses. Another epidural bolus consisting of 10 mL of 0.25% bupivacaine plus 100 mcg of fentanyl managed to briefly control her pain. At this time, examination revealed sensory block up to T6 but the patient was still complaining of sharp pain in her lower abdomen.

Due to being in labor induced by oxytocin for almost 24 hours and an arrest of active phase of labor at 6 cm cervical dilation for more than 6 hours with adequate contractions, the decision was made to proceed with primary cesarean section. The anesthesia team elected not to use the epidural due to its inadequate pain relief and performed a spinal anesthetic in the operating room. A single shot spinal was placed between the L1 and L2 interspace using 13.5 mg of bupivacaine 0.75% with 200 mcg of epinephrine and 200 mcg of preservative-free morphine and 25 mcg of fentanyl. The patient was still able to move her lower extremities and did not have adequate sensory or motor blocks for which she was put under general anesthesia. The procedure was uneventful and the patient emerged from anesthesia in a satisfactory condition and was placed on intravenous demand only patient-controlled analgesia.

**Figure 1:**
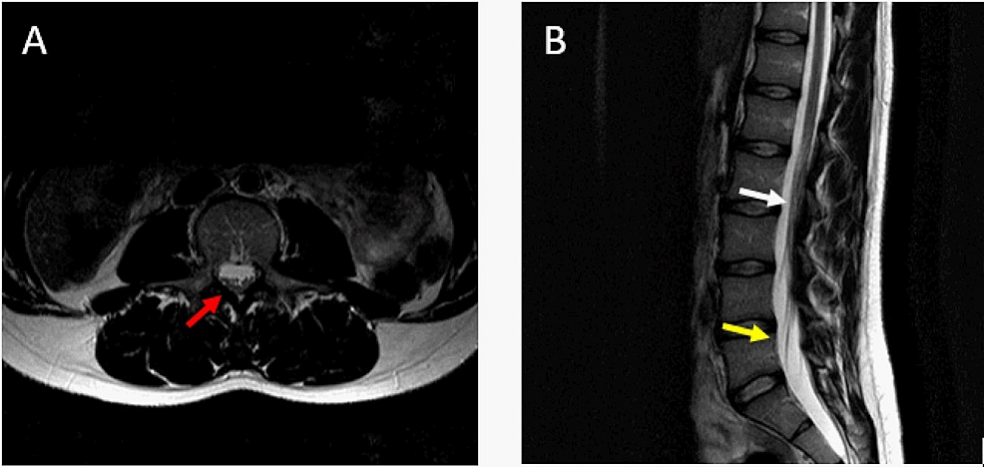
Patient’s lumbar MRI The first picture (A) corresponds to a transverse MRI view. We can observe fibrotic strands in the posterior aspect of the spinal cord (red arrow). Image B is a sagittal cut of the same MRI, the white arrow points to the section of the spine that is tethered, and the yellow arrow points toward the unusual low-lying cone.

An extensive chart review revealed a remote encounter with the spine health department several years before in a different hospital system in which she complained of lower back pain and weakness in her left foot. An MRI (Figures 1A-1B) revealed atypically low conus medullaris at the L3 level suggesting tethering.

## Discussion

Even though, as was previously described, spinal dysraphisms are not particularly common, they usually are not a complicated diagnosis. Most closed spinal dysraphisms, including tethered spinal cord, can be identified by multiple signs and symptoms: weakness, neuropathic pain, or paresthesia in the lower extremities, simple lower back pain, or lumbar or sacral skin abnormalities. The final diagnosis, however, must be established radiologically by demonstration of the spinal lesion. The optimal study for the characterization of intraspinal and perispinal anomalies associated with closed spinal dysraphism is an MRI of the entire spine. Treatment of these conditions branches in two main approaches, either a surgical correction or conservative monitoring of symptoms. Currently, there is not a consensus on the indication for surgery, but it is common practice to consider it for new-onset or progression of neurological symptoms. Severely affected patients with chronic, static symptoms don’t appear to benefit from surgical intervention [3].

Neuraxial anesthesia is especially popular for its capacity to bring total analgesia/anesthesia to the lower half of the body without the need for systemic medications. However, it strictly depends on the ability to reach the spinal segmental level that innervates the area of interest. Due to fibrotic, tethered tissue, medications delivered through the epidural catheter or spinal block were not able to spread completely and thus did not achieve a desirable analgesic level. Currently, there is not enough evidence to support a specific approach [4]. Multiple case reports have been documented regarding the complications of performing neuraxial procedures in patients with a tethered spinal cord, ranging from mild neurological symptoms [5] to permanent disability [6]. In general, the use of spinal anesthesia in patients diagnosed with closed spinal dysraphism is not recommended [4].

Another subject that needs to be addressed in this article is the fact that a previous diagnosis of the tethered spinal cord was apparently overlooked by the preoperative assessment consult. As demonstrated in this case, a comprehensive preoperative assessment is key to guarantee, to the fullest extent possible, an uneventful surgical procedure. The Preoperative Assessment and Optimization (PAO) is a common practice that has been refined over decades [7, 8]. Multiple studies have shown the importance of the PAO in terms of reducing unnecessary testing [9], case cancellations [10], and, more importantly, morbidity and mortality [11, 12].

Due to the fact that the patient in this case report omitted this information from the anesthesia team and also that the pathology was diagnosed and documented at an outside hospital system, this valuable information was not readily available for the anesthesia care team. All this finally resulted in the scenario where the clinicians in charge of her intrapartum analgesia were not aware of her tethered spinal cord.

## Conclusions

This patient had an active back pain problem, which had considerably affected her quality of life. This case highlights the importance and limitations of taking a thorough history, physical exam, and extensive chart review. We can safely say that all proper steps were followed during the pre-anesthetic assessment; the patient denied any medical problems and the electronic medical history was reviewed before placement of neuraxial anesthesia for labor. A possible recommendation would be to review not only previous notes but also any previous images/reports from outside hospitals to confirm the anesthetic plan.
